# Transgenerational role of seed mycobiome – an endosymbiotic fungal composition as a prerequisite to stress resilience and adaptive phenotypes in *Triticum*

**DOI:** 10.1038/s41598-019-54328-2

**Published:** 2019-12-06

**Authors:** Vladimir Vujanovic, M. Nazrul Islam, Prasad Daida

**Affiliations:** Department of Food and Bioproduct Sciences, College of Agriculture and Bioresources, University of Saskatchewan, Saskatoon, SK S7N 5A8 Canada

**Keywords:** Microbiome, Plant sciences

## Abstract

Illumina-MiSeq next-generation sequencing of ITS 5.8S rRNA gene demonstrated the transgenerational transmission of fungal seed-endophytes (mycobiome) across three consecutive wheat host generations under standard-control and drought conditions in the greenhouse. Drought-stressed plants experienced a positive shift in the seed mycobiome’s composition, moderated by the external acquisition of endophytic *Penicillium* (E+) at the seed level. Untreated (E−) and unstressed plants harbor a maximal fungal diversity of non-equilibrium ecological communities. While fungal composition in drought-stressed E− plants experienced important fluctuation, E+ plants maintained fungal ecological communities in phase equilibrium across generations. E+ plants hosted a relatively higher abundance of *Ascomycota* in the 2^nd^ and 3^rd^ seed generations of wheat, whereas higher abundance of *Basidiomycota* was detected in 1^st^ generation seeds. The dynamic response of ecological communities to environmental stress is conducive to E+ plants’ active recruitment of endosymbiotic consortia in seeds, benefiting host stress resilience and phenotype. In contrast, E− plants showed an erratic distribution of detected OTUs with an increased occurrence of phytopathogens and diminished plant performance under stress. The present study gives insight into the understanding of the seed-mycobiome composition and dynamics with the potential to improve plant host traits in an adverse environment.

## Introduction

The seed is considered as a plant reproductive unit which harbors a diversity of fungal endophytes^1^. Seed-associated fungal microbiomes (mycobiomes) are ubiquitous and thus potentially important for plant growth and sustainable crop production^2^. Although there has been a wide acceptance of the beneficial role of plant-root endophytes in enhancing plant defense and host growth promotion systems, seed-borne endophytes have been poorly explored^2,3^. Recent studies have found that mycovitality or the endosymbiotic seed–fungus relationship can improve stress resilience in addition to providing prenatal care to plant^3^ via coleorhiza (initial root)-specific dormancy release and enhanced biological seed stratification^4^. Also, there is evidence that some seed endophytes can influence plant post-germination phenophases^3,5,6^ due to the presence of fungal microbiome. Hence, it is foreseeable that seed-fungal microbiome coevolution directed towards interactive plant physiology could influence the host’s growth and stress mitigation^7^ under global warming^8,9^.

Many fungi are seed-borne endophytes and plant pathogens, henceforth, the seed is considered as an important vector in transmitting mycobiomes influencing plant host evolution as well as crop domestication over the last 10,000 years^10^. Endophytic fungi reveal a broad variation in their mode of transmission from one host to another^2^ and allow vertical transmission from one generation to the next^11^. Thus, it becomes essential to generate new knowledge and provide deeper insights into the beneficial seed-borne mycobiome, as well as stress-mitigating mechanisms in symbiotic plants having a possible translational effect on crop productivity^7^. Frequent drought stress particularly limits the performance of agriculture crops^12^ that may also negatively affect fungal and bacterial composition in stressed plants^13,14^. However, little is known about how seed selects mycobiome communities to establish efficient symbiotic associations in securing seed germination, plant growth, and reproduction, particularly under climate change^15^. The plant’s growth and crop reproduction success might be the result of genotype adaptation and associated evolutionary traits modulated by frequent shifts in host-associated fungal and bacterial microbiomes^16^ under fluctuating environmental conditions.

Under continuous pressure of increasing climatic alterations, it becomes imperative to better understand plant-microbiome relationships^7^ and to unravel the role of fungal microbiomes in conferring plant protection against abiotic stresses. Particularly, drought is limiting climatic factor to plant physiology and adaptation that might be palliated by the external acquisition of fungal endosymbionts at the seed level. Fungal endosymbionts control seed vitality via mycovitality, improved hydrothermal time of germination and myco-stratification^4,5,17^ to initiate plant growth promotion (PGP) under stress conditions^3,18^. Hence, the fungal endophytes with PGP trait bear tangible benefits to plant host functioning^19^. However, it is still unknown whether reintroduced PGP can induce shifts in plant mycobiome composition to improve host resistance in coping with adverse changing climate^20,21^. Therefore, characterizing the innate seed-mycobiome of *Triticum* with and without the external (horizontal) re-acquisition of the PGP fungus under drought stress can be a crucial step towards the understanding transgenerational transmission of seed endophytes and host-fungi interactions under climate change.

While the factors that drive plant microbiome composition are now better understood at the host-root level^22^, a major challenge for seed microbiology is to link microbial community composition and dynamics with ecological functions. As many fungi are seed-borne, we hypothesized that native endophytic seed fungal microbiome might be greatly influenced by local-drought environment and biological-acquisition of new fungal endophyte into seed conditions. The goal of this study was to figure out whether the stress-induced seed mycobiome profile may be an important effector of the plant adaptive phenotypes relative to transgenerational seed fungal composition. The Next Generation Sequencing (NGS) technology was employed for mapping core seed endophyte mycobiomes in wheat including host-mediated and subsequent-generations mycobiome selection under drought stress modified by *Penicillium* inoculation into seed. To understand complex interactions among these factors in seed, three specific hypotheses have been postulated. Firstly, the early inoculation of beneficial fungal endosymbiont at the first-generation wheat seed could result in the dynamically equilibrated flow of mycobiome composition across generations. Secondly, the transgenerational re-establishment of the seed-associated fungal communities depicts important points in mycobiome selection under stress towards the climate-resilient plant. Thirdly, seed endophytic consortia could play multiple roles in plant growth and fitness including host phenotypic traits.

## Material and Methods

In this study, we used Illumina-MiSeq next-generation sequencing (NGS) of ITS 5.8S to identify core fungal endophytes in wheat seeds of plants subjected to drought stress after parental seeds (G1) have been inoculated with the plant growth-promoting *Penicillium* sp. SMCD 2318 strain^3,4^. This experiment was designed as a continuous assessment of three-generational seed samples originating from the same seed lot and plants grown under the same drought and ambient (standard-control) greenhouse conditions.

### Plant under stress and transgenerational endophytic fungi

The present study was carried out using G1 surface-sterilized seeds of AC Avonlea wheat (*Triticum turgidum* L. var. durum)- grown in a controlled CONVIRON^®^ PGR15 growth chamber (Controlled Environments Ltd., Winnipeg, MB, Canada) devoid of the endophytic-*Penicillium* sp. 2318 strain. The second- and third-generation experiments tested first- and second-generation seeds, respectively, without additional SMCD 2318 inoculation on G2 and G3 plants.

Endophytic strain SMCD 2318 was originally isolated from wheat *Triticum* growing in Saskatchewan, Canada and deposited in the Saskatchewan Microbial Collection Database (SMCD). This organism can be readily re-isolated frequently from surface-sterilized wheat (*T. aestivum* and *T. turgidum* L. ssp. durum) seeds and flowers (anthesis stage), and occasionally from roots and leaves, indicating that it colonizes the above- and below-ground portions of wheat, preferably seed. The SMCD 2318 belongs to the group of *Penicillium* typically isolated from wheat seed^3,23^ also having specific symbiotic traits such as dormancy release and plant growth promotion^3,4,18,24^ which is of particular importance to this transgenerational-drought related study. The SMCD 2318 was grown on PDA at room temperature in the darkness for at least 3 d prior to experimental use.

**Figure 1:**
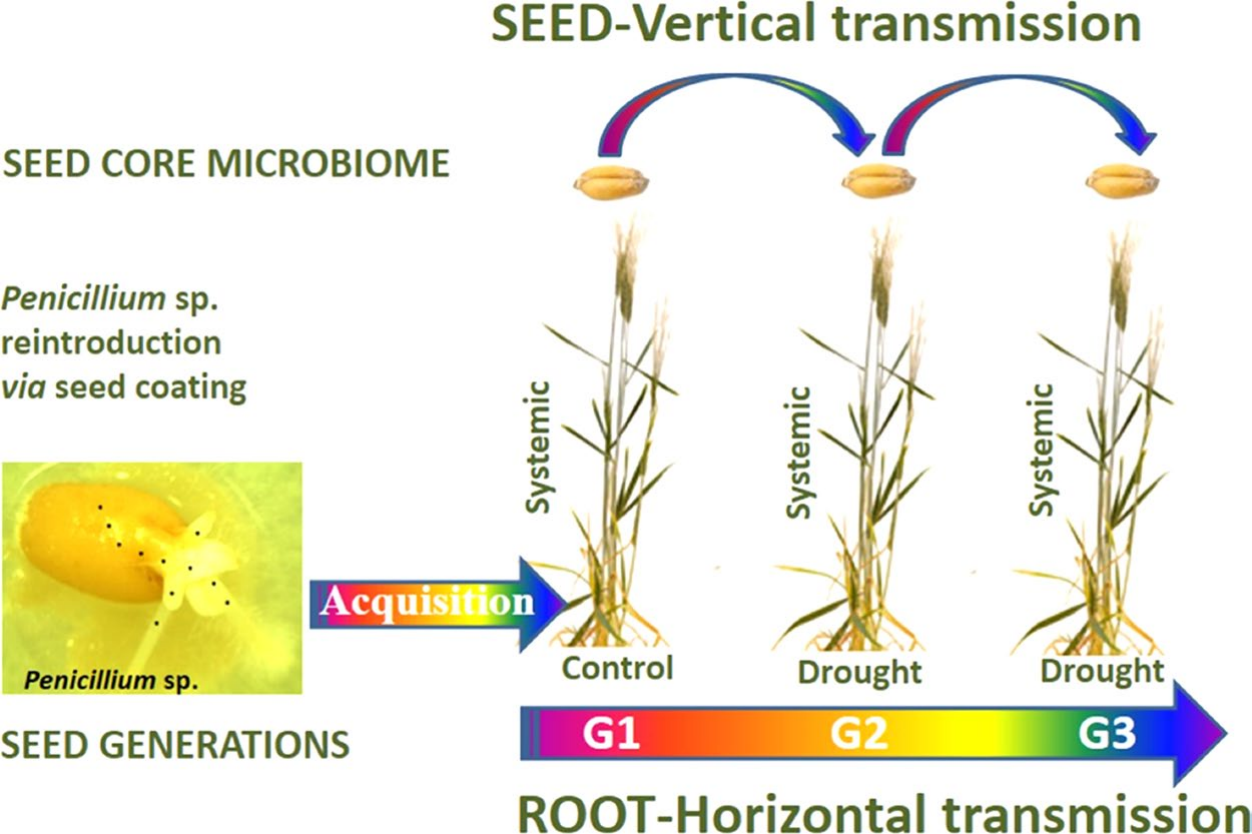
The flowchart shows the transgenerational (systemic, vertical, and horizontal) transmission of seed-associated fungal endophytes (mycobiome) over three consecutive wheat (*Triticum*) generations. The presence and transmission of seed-innate fungal endophytes were monitored using extracted fungal DNA from G1-uninoculated (E−), G1-inoculated (E+) seeds with *Penicillium* sp. (SMCD 2318) plant growth promoting inoculant*, and G2 (E−), G3 (E−) seeds as well. Seed germination, seedling growth and plant development in 4L pots were maintained under standard-control and drought stress conditions in the greenhouse. *Note: For details about the *Penicillium*-wheat seed interaction see Vujanovic et al. *Scientific Reports* 9 (2019) 1665 [ref.^58^].

In the greenhouse experiment (Fig. 1), the second (G2)- and third (G3)-generation seeds were produced from the *Penicillium* inoculated-first (G1)-generation seeds. It involved testing the effect of reintegration of the endophytic *Penicillium* strain into seed on the G1 level only followed by vertical (unlimited) and horizontal (pot-limited) transmission of microorganisms including a newly reintroduced SMCD 2318 from mother G1-seeds to G2- and G3-seed generations under drought stress. A second-generation experiment involved testing of first-generation seed, while third seed generation experiments involved testing of second-generation seeds without any additional SMCD 2318 inoculation on G2 and G3 plants. Treatment factors were three seed generations (G1-G2-G3) × four environments (standard-control, endophyte inoculation, drought-stressed, and drought stress with endophyte inoculation). In total, 12 treatments were carried out, with three replicates of each treatment using the randomized complete block (RCB) design. RCB is a recognized approach for multiple-generation seed viability trials in greenhouse^24^. Standard-control plants were grown separately under greenhouse conditions. The vertical microbial transmission was unlimited as linked to cross-pollination and microbial cell air transportation from plant to plant, while the limited horizontal root-root transmission was reduced on plants grown in the same pot. However, the possibility of the systemic microbial root to stem to seed transmission could not be excluded (Fig. 1).

All potted plants were grown in disinfected and separate greenhouse growth chambers from any other plant hosts. Seeds from three-generations were surface-sterilized in 75% ethanol for 10 s, rinsed with sterile distilled water for 10 s, submerged or 3 min in 5% sodium hypochlorite (Javex® 12 Bleach) and then rinsed five times with sterile distilled water^4^. For each treatment, five seeds were placed in one 4L plastic pots containing 400 g (dry weight) of autoclaved field capacity Sunshine mix 4 (SunGro Horticulture Canada Ltd., Vancouver, BC, Canada) consisting of three replicates. Wheat G1-seeds were surface-sterilized and coated with 48 h grown fungal endophyte SMCD 2318. Then covered with ~4 cm layer of sterilized Sunshine mix 4^24^ thus creating a spermosphere of the active soil zone for seed-fungus interaction^3^.

Drought stress was induced in the Plant Science greenhouse facilities at the University of Saskatchewan. In this experiment, the pots were arranged in a randomized complete block design (RCBD). Locations of blocks of pots and/or individual pots were changed every 2 weeks or more frequently. The daytime temperature ranged from 21 to 33 °C and overnight from 17 to 19 °C. Relative humidity ranged from 50 to 90%, with the lower and higher values occurring in the late afternoon and overnight, respectively. On sunny days, natural sunlight provided irradiation. On cloudy or winter days with reduced daylight photoperiodic conditions, 1000-watt high-pressure sodium light bulbs supplemented sunlight. These bulbs were suspended from the ceiling roughly 2 m above the plants. Standard-watered plants were kept at about 90–95% water-holding capacity (max. 100% only in time of watering), while drought-stressed plants grown at 25–30% soil water content by weight. During the experiment, an acclimatization period of one week commenced after seedlings reached Zadoks stage 12^25^ in 10 to 14 days. During acclimatization, the volume of water added to each pot daily was decreased by 50 ml per pot per day, from 300 ml per pot per two days down to 100 ml. Plant stress tolerance evaluated by quantifying replicated (n = 4) plant dry biomass (above ground tissues) samples and associated phenotypical changes in endophyte-inoculated (E+) and endophyte-free (E−) wheat plants exposed to drought stress and standard-control condition^24^. Seeds produced in the G1-generation were used without inoculant to generate G2- and G3-seed generations associated (vertically and horizontally) with transferred mycobiomes.

### PCR amplification and library preparation for high-throughput Illumina sequencing

In the present study, the three extracted DNA sub-samples were used for each treatment [3 DNA samples from 10 seeds/plant] followed by pooling the equal DNA ratio for three subsamples, so that a single DNA-composed sample consists of pooled DNA from 30 seed for each individual treatment. Genomic DNA of G1-G2-G3 seed samples [30 randomly selected seeds (3 × 10 seeds/plant) per each treatment] was extracted using DNeasy plant mini kit (Qiagen Inc. Canada) according to manufacturer’s instructions. Library preparation and Illumina sequencing were conducted at McGill University and Génome Québec Innovation Centre, Quebec, Canada.

The ITS 5.8S rRNA gene was amplified using forward primers containing the sequence TCGTCGGCAGCGTCAGATGTGTATAAGAGACAGCTTGGTCATTTAGAGGAAGTAA and reverse primer sequence GTCTCGTGGGCTCGGAGATGTGTATAAGAGACAG GCTGCGTTCTTCATCGATGC. The PCR amplification reactions were as followed: initial temperature at 96 °C for 15 min, for 33 cycles: 94 °C for 30 sec, 52 °C for 30 sec, 72 °C for 60 sec and final extension at 72 °C for 10 min, Besides the ITS target-specific sequence, adaptor sequences were added with primers allowing uniform amplification of the library with high complexity ready for downstream NGS on Illumina MiSeq. A subsequent limited-cycle amplification step was performed to add Illumina barcode sequences with primers and PCR amplification reactions as followed: initial temperature at 95 °C for 10 min, for 15 cycles: 95 °C for 15 sec, 60 °C for 30 sec, 72 °C for 60 sec and final extension at 72 °C for 3 min.

DNA quality was checked on a 0.8% agarose gel and DNA samples were quantified using a Qubit^®^ 2.0 Fluorometer (Invitrogen, CANADA). Between 5 and 50 ng DNA was used to generate amplicons using a Nextera^®^ XT DNA library preparation kit (Illumina Inc., USA). Libraries were normalized, pooled, and sequenced on the MiSeq platform. DNA libraries were validated using an Agilent 2100 Bioanalyzer (Agilent Technologies, USA) quantified by Qubit® and real-time PCR (Applied Biosystems, USA, and Bio-Rad, CANADA). DNA libraries were multiplexed and loaded on an Illumina MiSeq instrument according to the manufacturer’s instructions. Sequencing was performed using a 2 × 300 paired-end (PE) configuration; image analysis and base calling conducted by the MiSeq Control Software (MCS) on the MiSeq instrument. The primers used for sequencing were Read1: ACACTCTTTCCCTACACGACGCTCTTCCGATCT and Read2: GTGACTGGAGTTCAGACGTGTGCTC TTCCGATCT.

### ITS Illumina sequence data processing and phylogenetic analysis

The ITS-MiSeq raw sequences were processed by GENEWIZ® (www.genewiz.com). The percentage of bases with a quality score (Q-score) of 30 or higher were considered for downstream analysis. Denoising of pair-end MiSeq sequences-including demultiplexing; removal of low-quality reads and chimeras; and trimming of adapter sequences– was performed using the QIIME-pipeline^26^. The Metagenome@Kin Ver 2.0 (World Fusion, Tokyo, Japan) was used for phylogenetic classification of ITS-targeted amplicon reads using an Illumina-curated version of the UNITE taxonomic database equipped with a BLAST interface and assembled into Operational Taxonomic Units (OTUs) clusters at 97% identity. The application provided taxonomic names up to the taxon rank^27^, also, generating data at multiple lineage-family levels^28^.

The *Penicillium* SMCD 2318 endophytic isolate is easily culturable on potato dextrose agar (PDA; Difco, Detroit, MI, USA) in the absence of a host plant. Thus, the ITS-MiSeq Illumina sequencing detected SMCD 2318 presence in seed was further confirmed via re-isolation from surface-sterilized (G1, G2, and G3) seeds by Vujanovic *et al*.^23^.

### Statistical analysis of fungal diversity, sequence reads, and wheat biomass

The genetic diversity, richness, and evenness of seed associated fungal communities based on the number of operational taxonomic units (OTUs) were calculated^29–31^. The compositional variation among fungal communities was depicted using Shannon index for alpha diversity^29^ and Bray-Curtis dissimilarity ordination analysis for beta diversity^30,31^. The Shannon’s diversity index (*H*) that accounts for both abundance and evenness/equitability (*E*_*H*_) of the OTUs belonging to specific phylogenetic taxa present in three-generation seeds under standard-control, drought, inoculation, and the combination of drought and inoculation treatments. The proportion of the individual fungal taxa relative to the total number of taxa was calculated and then multiplied by the natural logarithm of this proportion. The resulting product is summed across species/taxa and multiplied by −1. Shannon’s evenness assumes a value between 0 and 1 with 1 being complete evenness.

The multivariate statistical package PC-ORD Vol.7. was used for hierarchical clustering dendrogram-heatmap at fungal family level and joint-plot principal component analysis (PCA) based on Euclidean distance matrix of the fungal OTUs classified at four fungal phyla (e.g. *Ascomycota*, *Basidiomycota*, *Chytridiomycota*, and *Zygomycota*) and *Incertae sedis*/unclassified in data matrix 1 and indices of diversity (i.e. Shannon, richness and evenness) in matrix 2 associated with 12 (3 generations × 4 environments) treatments. Dendrograms were computed using unweighted pair grouping by the arithmetic mean (UPGMA). All these statistical ordinations displayed abundance, distribution of fungal sequences (OTUs detected in the samples), and relationship with three-generation wheat seeds under four environmental exposures; drought, inoculation (E+), drought + inoculation, and standard-control (E−).

Sequence reads were subjected to a normality test performing skewness and kurtosis prior to analysis. The frequency distribution pattern of the sequence reads was designed under Gaussian bell-curve distribution model which has stretched by a factor sigma (σ) (standard deviation), and mu (µ) (arithmetic mean)^31^. The mean and deviation values of fungal sequence reads in the OTUs calculated from four environmental seed samples combining three-generation (G1-G2-G3) data. The ternary plots of fungal communities were conducted in R v.3.3.1 (R Foundation for Statistical Computing; available at http://www.R-project.org) using the *ggtern* package (version 2.1.4).

Ternary plots of the fungal composition based on top OTUs were conducted in R v.3.3.1 (R Foundation for Statistical Computing2) using the ggtern package (version 2.2.0). The relative abundance of the fungal OTUs classified as mold, pathogens and biocontrol was calculated as the percentage of sequence reads belonging to the specific OTU in relation to the total number of reads in a sample. Chart graph of the relative abundance of mold, pathogens and biocontrol in each treatment was created using Microsoft Excel 2016.

Wheat dry biomass of three-generations was assessed using two-way analysis of variance (ANOVA) considering generations (factor 1) and treatments (factor 2) as a source of variation using SAS v.15.1. (SAS Institute Inc. NC, USA). Post-hoc Tukey’s honest significant difference (HSD) test was performed to compare means of the four treatments within each of the generation at *P* ≤ 0.05.

## Results

These NGS-based ITS sequences discriminated core fungal endophytes in seeds of wheat plants subjected to drought stress after inoculation of parental seeds with the plant growth promoter *Penicillium* sp. SMCD 2318.

### Phylogenetic branching of seed endophytes

**Figure 2:**
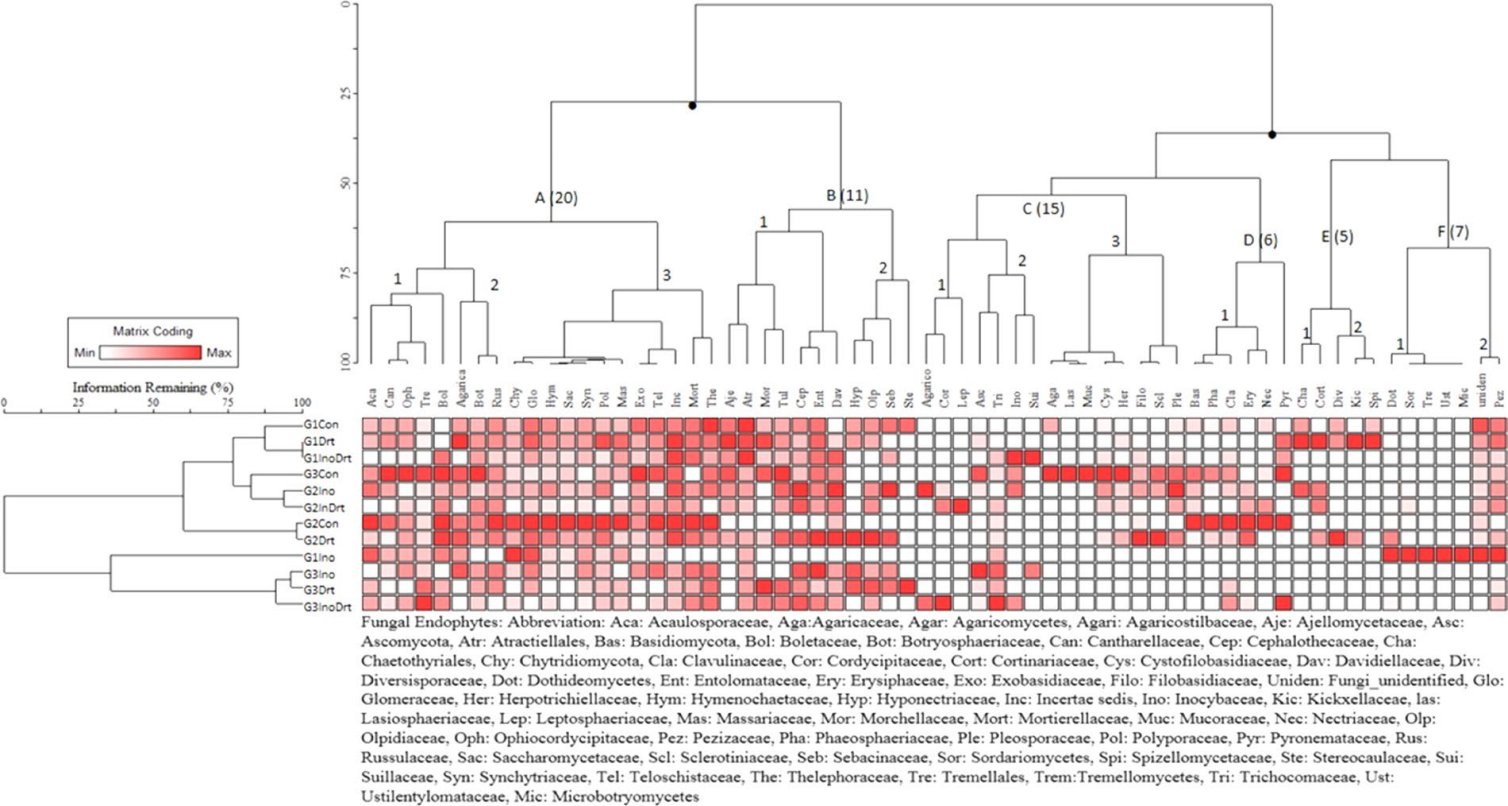
The effect of phylogenetic (family level), hierarchical clustered heatmap based on Euclidean distance between two points which depicts the abiotic (drought) stress on mycobiome abundance and composition in harvested standard-control (E−), inoculant (E+), uninoculated drought (E−) and drought plus inoculant (E+) seeds across three consecutive generations. Note: Con: Standard-Control, Drt: Drought, InoDrt: Inoculant + Drought.

The two-way clustered phylogenetic analyses generated hierarchically clustered heatmap (Fig. 2) which shows both abundance and distribution of fungal endophytic communities in wheat seed. All detected mycobiome communities were clustered in E+ and E− plant hosts grown under standard-control versus drought conditions across three consecutive wheat generations.

The endophytic *Penicillium* inoculant belongs to C (2)-*Trichocomaceae* group which represented by a total of 1890 sequence reads (data not shown) distributed in all the treatments across the generations. The C (2) was abundantly present in third generation (G3) inoculant (E+) and inoculant plus drought exposed seeds, whereas *Trichocomaceae* was completely absent in G1 standard-control and drought exposed seeds. However, G1-G2-G3-generation seeds had a lower abundance of *Trichocomaceae* regardless of treatments (Fig. 2).

The F (1) (i.e. *Dothideaceae*, *Sordariaceae*, *Tremellaceae*, *Ustilentylomataceae*) and F (2) (i.e. Unidentified, *Pezizaceae*) groups were high in abundance in inoculated (E+) G1-generation seeds (Fig. 2). However, G1- and G2-generation seeds under standard, inoculant, and drought conditions had lower abundance of F (2) groups, whereas, F (2) were greatly absent in G3-generation seeds regardless of treatments. On the other hand, A and B clusters formed a community of predominant^16^ fungal families commonly associated with all three-generations regardless of treatments with a few exceptions (Fig. 2). For example, A (1) groups were completely absent in inoculated drought G1 seeds, B (1) and (2) were absent or rarely occurred in G2-standard-control and G1-inoculant plus drought exposed plants, and A (3) was low in abundance or absent in the G3-generation seeds while inoculated plants grown under drought condition. Several interesting fungal distribution patterns were detected in drought stressed plants. For instance, *Agaricostilbaceae* groups were completely absent in all three-generation (G1-G2-G3) of seeds when inoculated (E+) plants exposed to drought. In contrast, *Cordycipitaceae* groups were abundantly present in the G2- and G3-generation of seeds when drought stressed plants were inoculated (E+) with *Penicillium* sp. SMCD 2318 (Fig. 2).

A few fungal taxa were clustered in A and B with low occurrence in G1-G2 standard-control seeds (Fig. 2). The C (1 and 2) groups of fungi were completely absent in G1-G2-generations under standard-condition, while they were dominant in G1-G2-generation of seeds under inoculant plus drought stressed conditions. However, G3-standard-control seeds harbored a higher abundance of C (2) over A and B group of endophytes (Fig. 2). Consequently, a profound increase of Shannon diversity index was revealed in standard-control seeds under G3-generation (Supplement Fig. 1S). The combination of environmental (abiotic and biotic) factors created a selective pressure thus allowing only specific fungal taxa or endophytic consortia within to be recruited from the whole mycobiome composed of *Ascomycota* and *Basidiomycota* followed by *Zygomycota*, *Chytridiomycota* and *Incertae sedis*/unclassified group of fungi. *Ascomycota* and *Basidiomycota* were the most abundant phyla across the treatments and generations (G1-G2-G3) (Supplement Fig. 2S).

### The bell-curve distribution model of seed mycobiome communities

**Figure 3:**
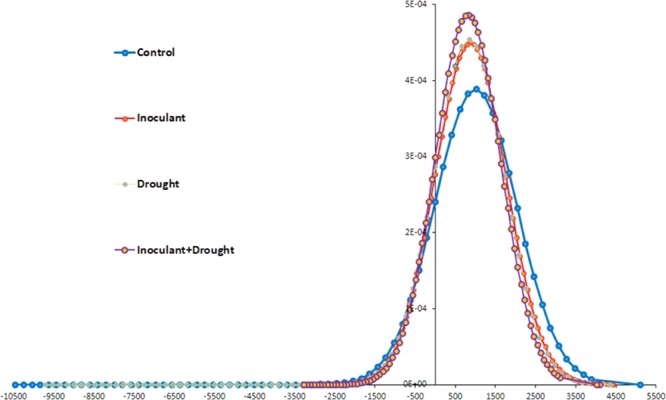
The frequency distribution model of the seed endophytes based on MiSeq ITS 5.8S sequences. Calculated distribution curves depict the capacity of seed-mycobiome communities and consortia to respond to external acquisition of the reintroduced endophytic fungal inoculant (*Penicillium* sp. SMCD 2318) and abiotic (drought) stress over three seed generations.

The Gaussian bell-curve, *a priori* pattern of distribution of the seed native fungal communities, made possible the identification of differences between treatments and presumed fungal taxa inheritances throughout seed transgenerational continuum. The curve revealed an unequal frequency distribution of the fungal endophytic sequences in wheat seeds associated with four treatments conditions (combination of three-generations) (Fig. 3). It is apparent that the distribution area (arithmetic means and standard deviations) decreased on the right side and increased on the left side corresponding to the positive vs. negative values under inoculant, drought, and inoculant plus drought compared to standard-control conditions (Fig. 3). The shift in distribution allows the pattern differentiation in endosymbiotic mycobiome assemblages exposed to biotic-inoculant and abiotic-drought stress, respectively. Despite the reduced frequency of the fungal sequences under standard-control condition, the higher mean value of sequence reads in standard condition (1066.4) negatively skewed (departure from horizontal symmetry) whereas, lower mean values in drought (844.6), inoculant (876.2) and inoculant plus drought (807.4) positively skewed over the frequency distribution curve. The drought exposed plants with and without endophyte treatment moved toward asymmetric left-curve while endophyte- and drought-free standard-control plants exhibited a normal-symmetrical distribution of quantities around a midpoint when the median approximates the mean (Fig. 3).

### Dynamics of seed endophytes over generations

**Figure 4:**
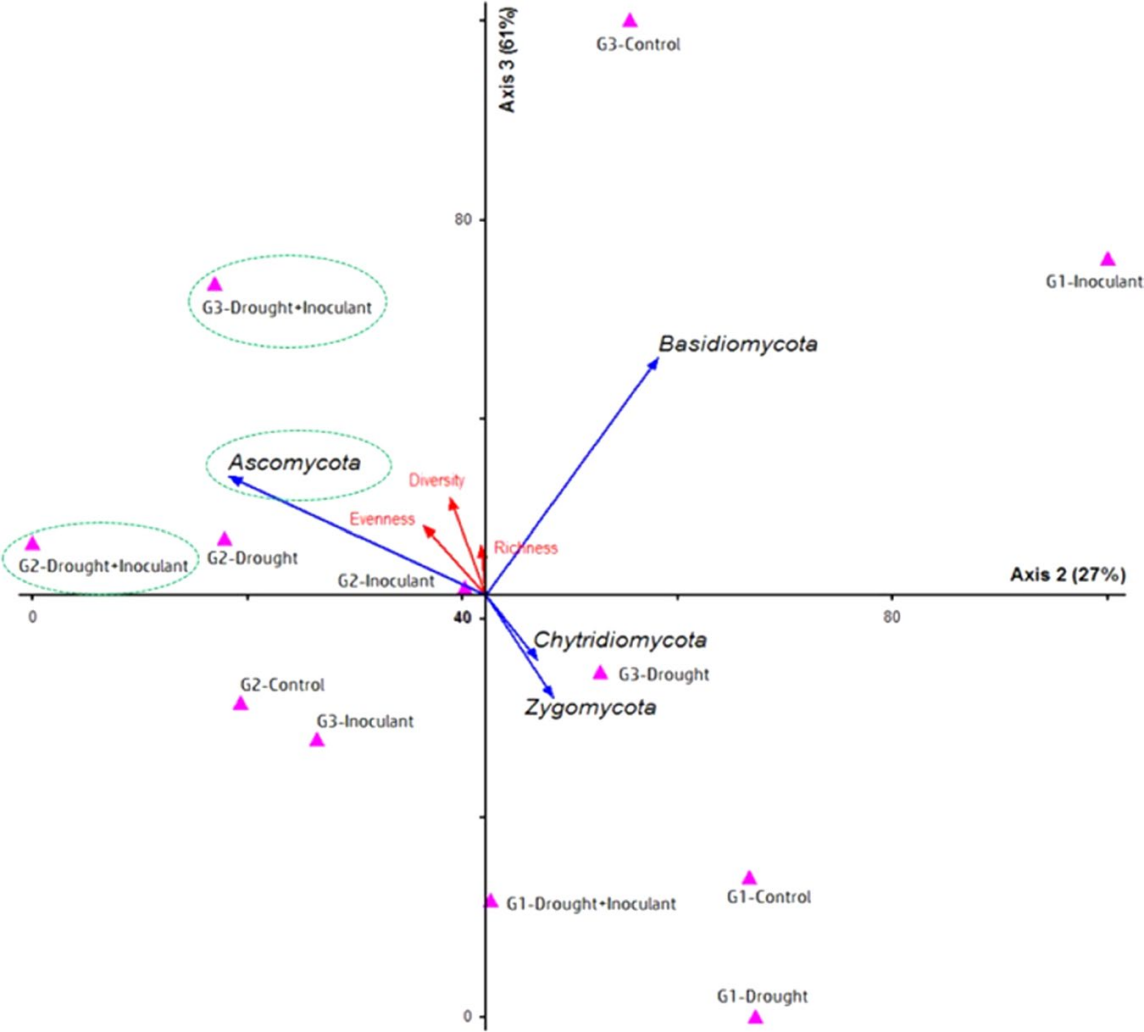
The principal component analysis (PCA)^62^ based on Euclidean distance matrix showing the relationship between two-plot data matrics. Matrix 1 consists of the sequence abundance (ITS 5.8S gene) of fungal phyla-*Ascomycota*, *Basidiomycota*, *Chytrideomycota*, and *Zygomycota* and diversity (Shannon-Weiver), richness, and evenness of fungal communities in matrix 2 associated with three generation (G1, G2, and G3) of wheat seeds under standard-control, drought, inoculant, and drought + inoculant treatments. The direction and length of the radiating blue (fungal phyla) and red (diversity indices) lines indicating the strenght and the degree of relationship among the treatments over geberations. The dash circles pointing the most influencial fungal phylum on the two treatment variables.

The PCA based on Euclidean distance matrix had three dimensions solution, the best two explaining 27% variance on axis 2 and 61% on axis 3 of fungal community composition (Fig. 4). This joint-plot ordination demonstrated the relationship between the abundance, Shannon diversity, richness, and evenness indices of seed associated fungal endophytes over three-generation (G1-G2-G3) under inoculant and drought interventions. The angles and length of the radiating lines indicate the strength and relationships of the variables such as the Shannon diversity and evenness. These indices had a greater influence on the composition and distribution of *Ascomycota* in E+ plants with or without drought exposures under two later (G2 and G3) generations of seeds, compared to other fungal groups (Fig. 4). However, few events such as G1 (control, drought, and drought plus inoculant) and G2 (control), and G3 (drought) had strong influence on higher diversity and evenness of *Chytridiomycota* and *Zygomycota* groups, compared to diversity and evenness of *Ascomycota* and *Basidiomycota* groups over the generations and treatments (Fig. 4).

The maximum evenness values (0.36 and 0.37) of fungal endophytes were recorded in G2 seeds after acquiring *Penicillium* sp. via inoculation during drought or without drought exposures (Supplement Fig. 1S). As a result, homogeneous distribution of endophytic sequences over G2-generation under E+ plants in drought-stressed conditions (Supplement Fig. 3A–D; see small circles). In contrast, a considerable reduction in OTU richness, evenness and diversity from G2 to G3 generations was detected in E+ plants under drought-stressed conditions (Supplement Fig. 1S). Interestingly, the richness in E+ plus drought exposed seeds remained equilibrated as relatively static or unaffected over G1 and G2 generations. Similarly, Shannon diversity index was consistently higher in drought-stressed G2 seeds where plant received *Penicillium* sp. SMCD 2318 strain (Supplement Fig. 1S).

*Penicillium* inoculation had a positive impact on *Ascomycota*, as an increasing trend of its OTUs abundance over three-generations (Supplement Fig. 2S). Whereas both standard-control and drought stressed conditions had a reduced number of *Ascomycota*, specifically in G1- and G3-generations. In contrast, *Basidiomycota* tends to increase their presence over three consecutive seed generations under inoculant-free conditions (control and drought) (Supplement Fig. 2S). The relatively low abundant endophytes, *Zygomycota*, and *Chytridiomycota* remained constant across the generations under four treatments condition. The abundance of *Incertae sedis*/unidentified fungal sequences was progressively reduced (1000 → 400) over the generations and the reduced trend was consistent in all treatments (Supplement Fig. 2S). Further, Supplement Fig. 3S depicts the effect of treatments on fluctuation of the relative abundance of predominant fugal OTUs across sample groups associated with G1, G2 and G3 generations. The fungal taxa in family *Pleosporaceae* showed drastic shift under drought, inoculant (E+) and inoculant-drought treatments in G1 and G3 generations compared with inoculant treatment under standard-control condition. Also, drought and drought-inoculant treatments were shifted most of the OTUs in *Trichocomaceae* and *Tremellaceae* associated to G3 plants as compared to standard-control plants (Supplement Fig. 3S).

In inoculant treated seeds (Supplement Fig. 4S), the relative abundance of the common wheat molds (*Aspergillus* in *Trichocomaceae* and *Alternaria* in *Pleosporaceae*) and phytopathogens (*Colletorichum* in *Glomeraceae*, *Giberella/Fusarium* in *Nectriaceae* and *Stagonospora* in *Pheosphaeriaceae*) was reduced, 250 → 40, 209 → 93 and 71 → 17, respectively, across generations. In the same inoculant samples, the biocontrol fungal consortium - *Acremonium/Sarocladium* and Hypocrea/Trichoderma belonging to *Hypocreceae* - gradually increased abundance over G1-G2-G3 generations, The *Giberella/Fusarium* reduced abundance also coincides with increased abundance of the biocontrol taxa; however, it was independent variable of the type of the treatment and the generation of the seed.

### Wheat biomass and phenotypic changes

**Figure 5:**
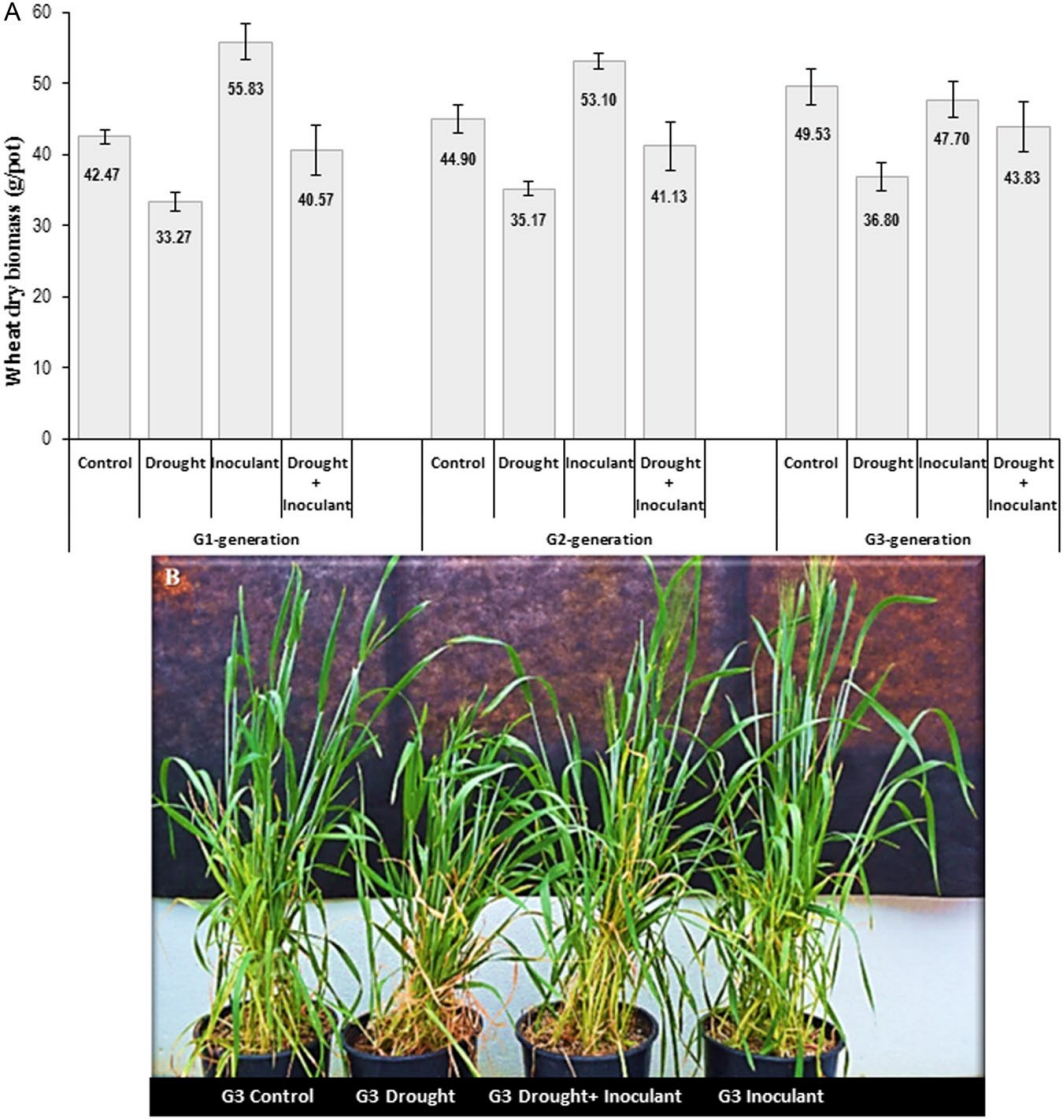
(**A**,**B**) The impact of drought and *Penicillium* sp. SMCD 2318 -PGP inoculant on (**A**). Wheat biomass across three seed generations using two-way statistical analysis of variance (ANOVA) with post-hoc Tukey’s honest significant (*P* ≤ 0.05) difference (HSD) test among the treatments at each of the three-generations, and (**B**). Phenotypic characteristics of G3 plants grown under four treatment conditions. Different letters within each generation indicate significant changes (*P* *≤* 0.05).

Seed mycobiome diversity and abundance fluctuated in response to the type of abiotic and biotic environmental stimuli. The phenotypic traits of the aerial plant parts (i.e. stem, leaf, tillers, and spikes/kernels), coincided with the shift in wheat host biomass over generations (Fig. 5A,B). Two-way ANOVA revealed that treatments (endophytic-inoculant and drought) under three-generations had significant (*P* = 0.023) impact on the accumulation of wheat dry biomass, however, the changes of biomass over the generations were non-significant (*P* = 0.023) (Fig. 5A). The biomass of uninoculated drought-stressed plants (E−) was significantly (*P* ≤ 0.05) reduced under all three-generations (G1-G2-G3). Endophytic-inoculant in standard-control plants (E+) showed significant (*P* ≤ 0.05) increase of biomass under three-generation compared to uninoculated drought-stressed plants (Fig. 5A). When inoculant added in standard-control plants, the biomass was significantly enhanced compared to uninoculated standard-control plants and this trend was consistent at all three-generations, however, addition of inoculant in drought-stressed plants, the biomass was significantly increased only in G1- and G2-generations, but not in G3-generation, compared to uninoculated standard control (Fig. 5A).

In drought exposed plants, the chronological phenotypic changes were equally observed (data not shown). It was apparent that the phenophases following seedlings emergence, including stem elongation and formation of tillers and spikes, were enhanced in endophyte-treated plants, which eventually shaped up a relatively bigger canopy in G3-generation compared to endophyte-free drought-stressed plants (Fig. 5B). Plants under drought stress, inoculated with endophyte were taller than inoculant-free (E−) drought-stressed plants (Fig. 5B). Consequently, in all G1-G2-G3 generations, significantly (*P* ≤ 0.01) improved biomass was achieved from plants associated with endophyte (E+) and without drought compared to endophyte-free (E−) drought-stressed plants (Fig. 5A).

**Figure 6:**
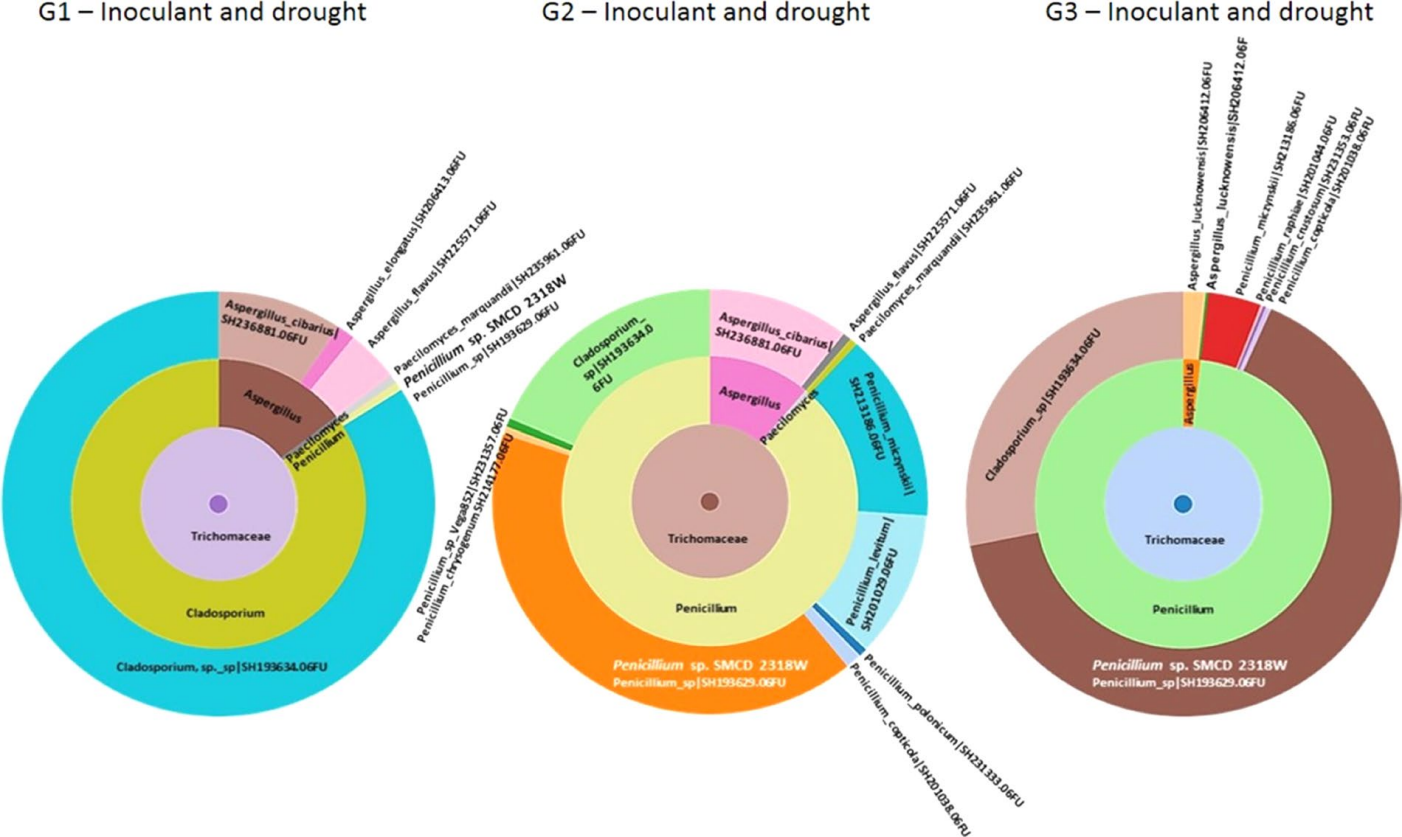
Sunburst-Illumina based chart showing two predominating *Trichomaceae* (*Penicillium, Aspergillus*, and *Paecilomyces* species) and *Davidiellaceae* (*Cladosporium* species) families over three consecutive generations of *Triticum*. The sequential reintroduction and establishment of *Penicillium* sp. SMCD 2318 strains in wheat seeds grown under greenhouse drought conditions.

The improved biomass observed in drought-stressed plants coincides with the successful reintroduction of *Penicillium* sp. strain in G1 plants (Fig. 6). Illumina-sunburst analysis exhibited that the *Penicillium* (*Trichocomaceae*) endophyte was predominant seed colonizer in G2-G3 plants under drought-stressed condition followed by *Cladosporium* (*Davidiellaceae*) species. The sunburst illustration also showed that other *Trichocomaceae* taxa, such as *Aspergillus* and *Paecilomyces* species, did not considerably change in abundance throughout G1-G2-G3 drought stressed-plants generations (Fig. 6).

## Discussion

The true motivation to study the seed-inhabiting mycobiome lies on the particular fungal traits to be transmitted vertically to the progeny in promoting host-specific germination and improved transgenerational host phenotypes. In the present study, we depicted some temporal dynamics of the seed-born fungal endophytic communities after drought disturbance events. It appears that the more resilient community recovers to its initial state after perturbation, particularly when the newly reintroduced fungal endophyte PGP -*Penicillium* was acquired into first-generation seed. The resilient fungal community members were recruited from the environmental/native microbial pool and so in a sustainable way across generations. The present study revealed the mycobiome transgenerational transfer as an integral part of the evolution of the seed-micro ecosystem. According to Orozco-Mosqueda *et al*.^32^, the predictability of the multi-generation inheritance of endophytic genome components in plants is one of the major challenges involved in the use of endophytes. In that regard, we discovered that if the plant growth benefits are provided early at the seed germination level by the endophytic inoculum, the resilient holobiome (a host plus its microbiome acting alongside as an ecological community of organisms) may still be able to enhance crop productivity under stress similar to unstressed plants across generations. To mitigate stress while not compromising plant growth and yield has been suggested to be an ideal strategy. To the best of our knowledge, this is the first report of the microbiome shift in the presence of endophyte with desired seed ecological characteristics showing the potentially significant impact on host phenotypical traits under drought stress. These findings reinforce the concepts of holobiont-mediated adaptation via host-mediated and microbiome-mediated selection processes working in a concert to better respond to external environmental factors. The modern concept of evolution considers holobiont and its hologenome-composed of plant genome and associated microbiomes^33^. Hence, multicellular organisms can no longer be considered individuals by the classical definitions of the term^34^. Microbial symbionts, including multicellular fungi, can be reinstalled or acquired by plant and transmitted from parent to offspring by horizontal and vertical pathways using vectors and the environment. Several studies have reported that these symbionts contribute to the anatomy, physiology, development, innate or adaptive immunity, and behavior^35,36^, thus leading to genetic variation, origin, and evolution of species^37^. It seems that the acquisition of microbes and microbial genes is a powerful mechanism for driving the evolution of complexity. Evolution of the human microbiome proceeds via both cooperation and competition working in parallel^38^, which could be a conceivable way to drive a structure of microbiomes in the plant. Indeed, the MiSeq Illumina sequence data depicted significant changes in both wheat resilience and phenotype/biomass which coincide with changes in the composition and diversity of the seed endophytic fungal communities.

In some instances, endophytic fungal symbionts undergo mixed horizontal and vertical transmission, also named an “imperfect transmission” when the symbiont is not transmitted to all offspring^39^. In this study, MiSeq-ITS sequences analyses depicted a highly dynamic process in fluctuating fungal endophytes over three seed generations. The results also revealed a continuum in the transmission of fungal communities from the first seed generation to subsequent second and third generations, also implying a selective systemic fungal transmission. Using the ITS sequence-based approach, Ganley and Newcombe^40^ were able to discriminate the transmission of diverse fungal endophytes in coniferous seeds.

The results of the present study revealed that seed-endosymbiotic fungal mutualism over the generations was greatly affected by both plant-*Penicillium* sp. acquisition and plant-drought exposure regimes. The diversity of seed endophytes under drought or shortage of water was mediated by the *Penicillium* inoculant. Notably, the occurrence of the *Trichomaceae* including *Penicillium, Aspergillus*, and *Paecilomyces* predominated over generations, particularly in inoculated drought exposed seeds. In contrast, *Trichomaceae* taxa were rare in first-generation standard-control seeds. According to Barret *et al*.^41^ a plant’s genotype has a strong effect on the dynamics of the seed microbiota. An increased presence of the *Penicilllium* inoculant on seed could induce a shift in concurrence of r- vs. K-strategists^42^ due to the possible effect of *Penicillium* on the fungal microbiota’s structure during seed germination and seedling emergence. This fungal inoculant has a fast-growing ability (r-strategist) that may explain its rapid colonization of seedlings.

Inoculated and drought exposed G1 seeds showed an initial presence of *Penicillium* inoculant, and the predominance of *Cladosporium* sp., a member of the *Davidiellaceae* (Fig. 6). The maximum shift of *Penicillium* over *Cladosporium* was detected in G2 drought-stressed generation. It resulted in re-equilibrated *Penicillium*:*Cladosporium* dynamic state associated with G3 drought-stressed generation. Despite that equilibrium, the diversity of *Penicillium* species increased from drought-stressed G1 (2-species including *Penicillium* sp.-inoculant) to drought-stressed G2 and G3 (7-species including *Penicillium* sp.-inoculant) generations. In the same time, the number of *Cladosporium, Aspergillus*, and *Paecilomyces* species remained stable, 1–2 taxa, throughout all three drought-stressed generations. This phenomenon supports the first hypothesis that the external inoculant recruitment could dictate a process of both endosymbiont transmission and retention thus equilibrating composition in the wheat seeds over generations. In addition, several studies showed evidence of vertical transmission of seed associated endosymbionts up to the second generation of the host from different plant organs via vascular connections or through gametes (systemic entry)^43–47^. Further, the seed allows coexistence of various fungal endopsheric consortia. When exposed to the environmental stress/stressors, the fungal taxa (species, guilds and communities) can alter in diversity and abundance. It also appears that the *Penicillium* inoculant can modulate abundance of different functional groups of seed-born fungi. Its increased abundance coincides with the reduction of certain common molds (*Alternaria and Aspergillus*) as well as phytopathogens such as *Colletotrichum* (anthracnose), *Fusarium* (crown rot and head blight) and *Staganospora* (leaf spot).

Seed is an environmental niche which creates conducive environment for hosting various functional groups of fungi. Nevertheless, the protective effect of the inoculant against molds/phytopathogens may be related to *Penicillium*’s competitive ability for occupying most of the seed microenvironments. In terms of the biocontrol consortia, *Acremonium*/*Sarocladium* and *Trichoderma* species appeared to gradually increase abundance from G1-inoculant to G3 inoculant treatment. The highest biocontrol abundance coincides with the lowest abundance of the *Giberella*/*Fusarium* across all generations. This finding may open a new avenue to better understand the influence of the endophytic inoculant on seed mycobiome as well as transmission and persistence of the fungal species, and consortia from generation to generation. Subsequently, results may encourage plant microbiologists to work more closely with plant ecologists, pathologists and breeders to take advantage of these findings.

Wheat preferentially chose distinct endophytic taxa into seeds depending on the type of the environmental stresses. Kingdom Fungi provided all phyla with a predominance of endophytic *Ascomycota* and *Basidiomycota* occupying wheat seed followed by *Incertae sedis*, *Chytridiomycota*, and *Zygomycota*. *Chytridiomycota* and *Zygomycota* represent the earliest terrestrial divergences from ancestral aquatic fungi^48^. Typical environmental conditions, optimal for plant growth, dictate normal or Gaussian fungal distribution in the seed. Only PGP-inoculum treatment combined with drought stress continuously increased the presence of *Ascomycota*. This phylum showed an increased cumulative proportion over time, while *Basidiomycota* preferentially displayed increasing proportion and so exclusively related to the combination of inoculant and drought conditions. The endmost indicates the existence of the three most equilibrated states in dynamic of seed-mycobiome communities, named “standard”- control, “induced”- drought stress alone or inoculant acquisition alone, and “combined” inoculant plus drought intervention. The dynamic changes in both fungal richness and evenness induced by “combined” intervention seemed to induce a substantial shift in host-mycobiome interactions to reinstate host resilience and phenotypic trait. The induced stresses followed by the alteration in fungal phylogenetic groups from the *r*-growers to the K-growers^49^ also indicate a possible fluctuation in seed nutrients throughout generations. It has been reported for human gut microbiome that fungal diversity correlated with diet, nutrient values in the habitat, as well as associated bacterial inhabitants^50^.

The fungal distribution in standard-control plants served as initial diversity or a pool of non-equilibrated microbial communities dispersed over generations. Imposed drought stress and symbiont inoculation moved the overall system toward asymmetric left-curve in a new preferential equilibrium (Fig. 3) and thus a more desirable system for seed when exposed to the biotic or abiotic external influences. Consequently, the asymmetric movement of the sequence values in seed endophytes influenced by biotic and abiotic factors was presumably correlated with the abundance of two fungal E (1 and 2) and B (2) clusters or communities (Fig. 2). They were detected in G1-G2-inoculant and inoculant plus drought exposed seeds characterized by positive skewness fungal distribution from the normal-symmetry of the curve. Interestingly, standard-control seeds were not harbored the majority of those fungal taxa and its mycobiome’s curve followed a normal frequency distribution model. It indicates the shift in specific taxa selection occurred in response to the tested environmental influences. For instance, the members of family *Pleosporaceae* shifts in drought stress related diversity and abundance (Supplement Fig. 3S). This family contains both endophytic fungi and opportunistic plant pathogens, such as *Alternaria, Bipolaris,Curvularia, Exserohilum, Pleospora* and *Pyrenophora*, which requests further investigation and proper explanation^51^. Similarly, this can be applied on the members of family *Davidiellaceae*, i.e. genus *Cladosporium/Davidiella* might be considered as potential biocontrol (*C. cladosporioides*, C. *tenuissima* and *C. uredinicola*) against wheat diseases on the mature plants, although it is often categorized (as in this paper) as a potential mold on the seed level. Further, some F (1) and F (2) fungal groups were undetectable in G3-generation seeds (Fig. 2) regardless of biotic and abiotic influences as possibly stress-unspecific taxa. However, both specific and unspecific direction changes in the distribution of the fungal taxa may be the result of the cross-talk between fungi, microbes, and seed host (occupied plant ecological niche) towards phase equilibrium in mycobiome composition and dynamics over time. Hence, our second hypothesis supports the prediction of the changes in the outcome distribution of the fungal OTUs^52^, based on MiSeq Illumina NGS technology^53^ that facilitates combining information from phylogenetic branching events and bell-curves, as an effective means to find out how fungal communities behave to adapt and disperse over plant-seed generations.

In this study, the motivational direction of the third hypothesis was to verify the potential phenotypical changes in wheat during drought exposure with and without endophytic intervention. Current results indicate that the persistence of the introduced endophytic inoculant over three seed generations has an influence on the mycobiome composition towards an improved plant biomass production under drought conditions rather than standard-control growth conditions (Fig. 5). It seems that the phenotype of the symbiotic (E+) G1 plant response to drought persisted beyond the first seed generation. The phenotypic changes were typically associated with preferential endophytic consortia transferred via seed which can assist the altered wheat traits over generations. It implies that plants under stress may actively recruit members of the mycobiome community for steady state systems where substantial insight into energy balance and plant metabolic rates can be achieved^21^. Selection of suitable fungal endosymbionts^54–56^ promote plant phenotype and ameliorate resilience to water scarcity sustaining fungal ecological niche. Some possible mechanisms driving drought tolerance may be linked to reduced stomatal conductance^57^ and favorable biochemical processes leading to balanced water regime and equilibrated nutritional status of the plant^58–60^. Moreover, the observation by Molina-Montenegro *et al*.^61^ is in a partial agreement with our findings. They reported that the net photosynthesis, fresh and dry biomass production, as well as water use efficiency were significantly higher in the treatments with the presence of inoculated root-endophytes at 25% reduced water regimes, compared to endophyte-free lettuce cultivar. Further research in that direction on seeds from different host genotypes and throughout world’s ecoregions is highly merited. Our results provide important insights into the dynamics of seed endosymbionts over generations, as a possible regulatory mechanism of plant phenotypic adaptation and resilience under an external constraint.

In summary, the evolutionary mechanisms of seed microbiome distribution and dynamics can be exploited for the selection and implementation of endophytic microbial inoculants, specific to the plant genotype(s) and environmental condition(s). Some endophytes appeared to be commonly distributed, others limited to biotic and abiotic influences in one or more seed generations. The ITS 5.8S gene distribution curve model depicted a distinctive shift in a fine scale of the core seed mycobiome variations consisting of native fungal taxa. It allows appreciating an important and intriguing role of endophyte horizontal acquisition in seed via artificial seed coating further shaping the recruitment and dynamic of native endosymbionts over generations. The ever-changing dynamics of seed mycobiome seem to be driven simultaneously by inoculant and drought influences resulting in subsequent changes in plant phenotype and biomass production. However, actual mechanism and driving factors in inoculant transmission process over seed generations and subsequent role in triggering biomass accumulation under water-stressed plant tissue conditions were not clearly understood. The manipulation of the mycobiome by re-introducing an endophytic strain in plant system, such as endophytic *Penicillium* at seed germination stage^58^, is apparent. It provides an opportunity to promote specific consortia of fungal endophytes in seed as reproductive unit coping with climate change. The occurrence of the stress-induced mycobiome profile may be an important effector in reaching seed phase equilibrium maintained under drought stress across generations. Although no causal relationship between different observed parameters was fully established, a considerable correlation between the structure and dynamics of the seed-endosymbionts, transmitted fungal inoculant to seed and shifted phenotypic changes were unveiled. These changes resulted in improved plant biomass and resilience under abiotic stress.

In conclusion, the new seed mycobiome taxa discovered herein will spur further questions associated with the functional dynamics and genetic network of host-endophyte interactions under stressed conditions. Our current understanding-based on transgenerational assessment of seed-mycobiome relationships is built on research experiments under controlled conditions; it warrants further experimentation in field settings for a deeper insight into the practical aspects of sustainable food production and new breeding programs for symbiotic fungal endophytes in staple crops.

## Supplementary information

Supplementary figures F1S-F4S

## Data Availability

All data generated or analyzed during this study are included in this published article.
