# Supplementary material for: Transgenerational role of seed mycobiome – an endosymbiotic fungal composition as a prerequisite to stress resilience and adaptive phenotypes in Triticum
Source: Sci Rep. 2019 Dec 6;9:18483. doi: 10.1038/s41598-019-54328-2 (PMC6898677; doi:10.1038/s41598-019-54328-2)
Supplement: Supplementary file 1 — Supplementary figures F1S-F4S [file 41598_2019_54328_MOESM1_ESM.pdf]

**Supplementary Information**

**Article in Scientific Reports**

**Transgenerational role of seed mycobiome – an endosymbiotic fungal composition as a prerequisite to stress resilience and adaptive phenotypes in *Triticum***

Vladimir Vujanovic, M. Nazrul Islam, Prasad Daida

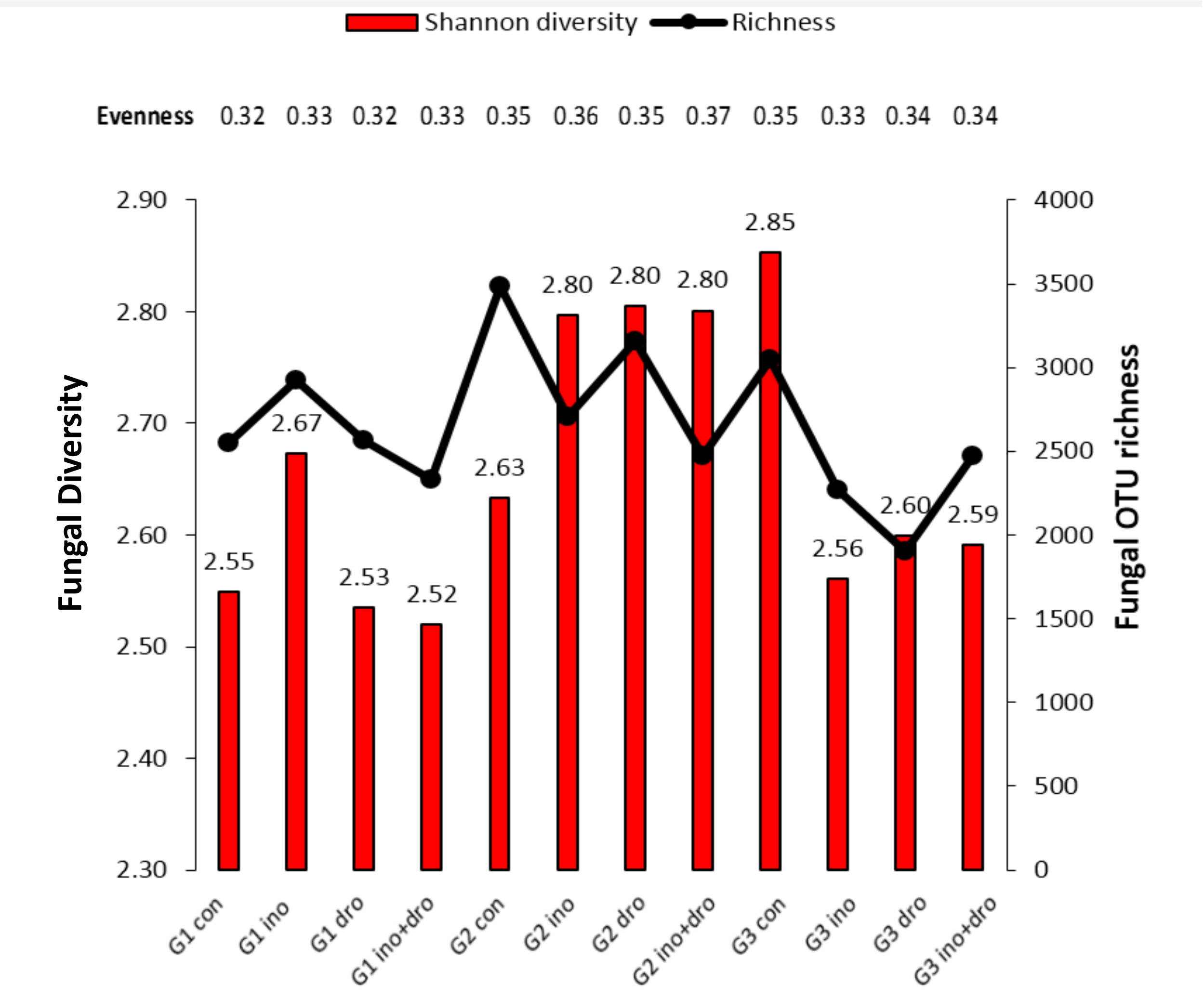


**Fig. 1S.** Shannon-Weaver diversity, OTU richness and evenness of fungal endophytes in three generation (G1, G2 and G3) of wheat seeds grown in glasshouse under standard-control (con), inoculant (ino), drought (dro) and inoculant plus drought (ino+dro) conditions.

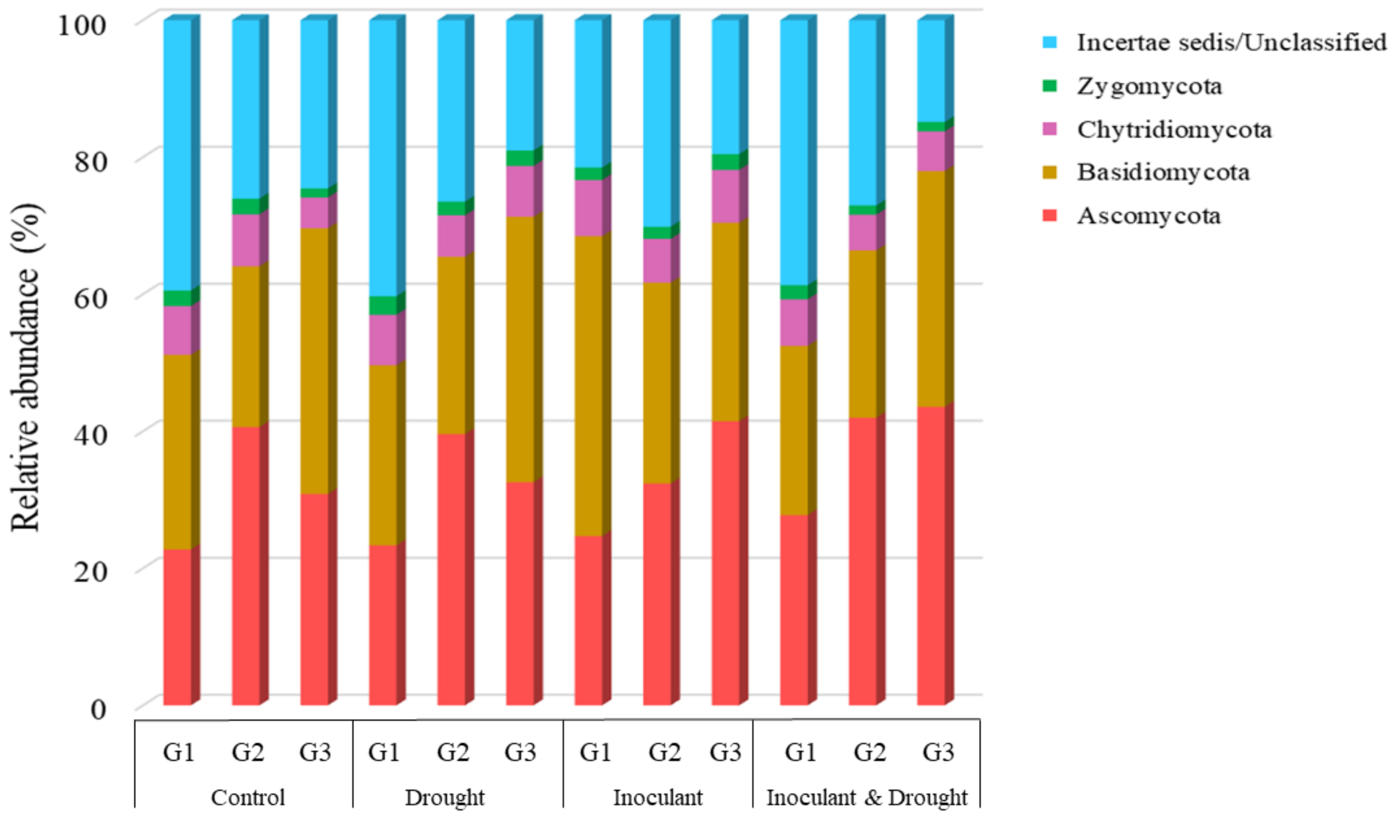


**Fig. 2S.** Relative abundance of endophytic fungal phyla associated with G1, G2, and G3 generation of seeds under standard-control, drought, inoculant, and inoculant plus drought treatment conditions.

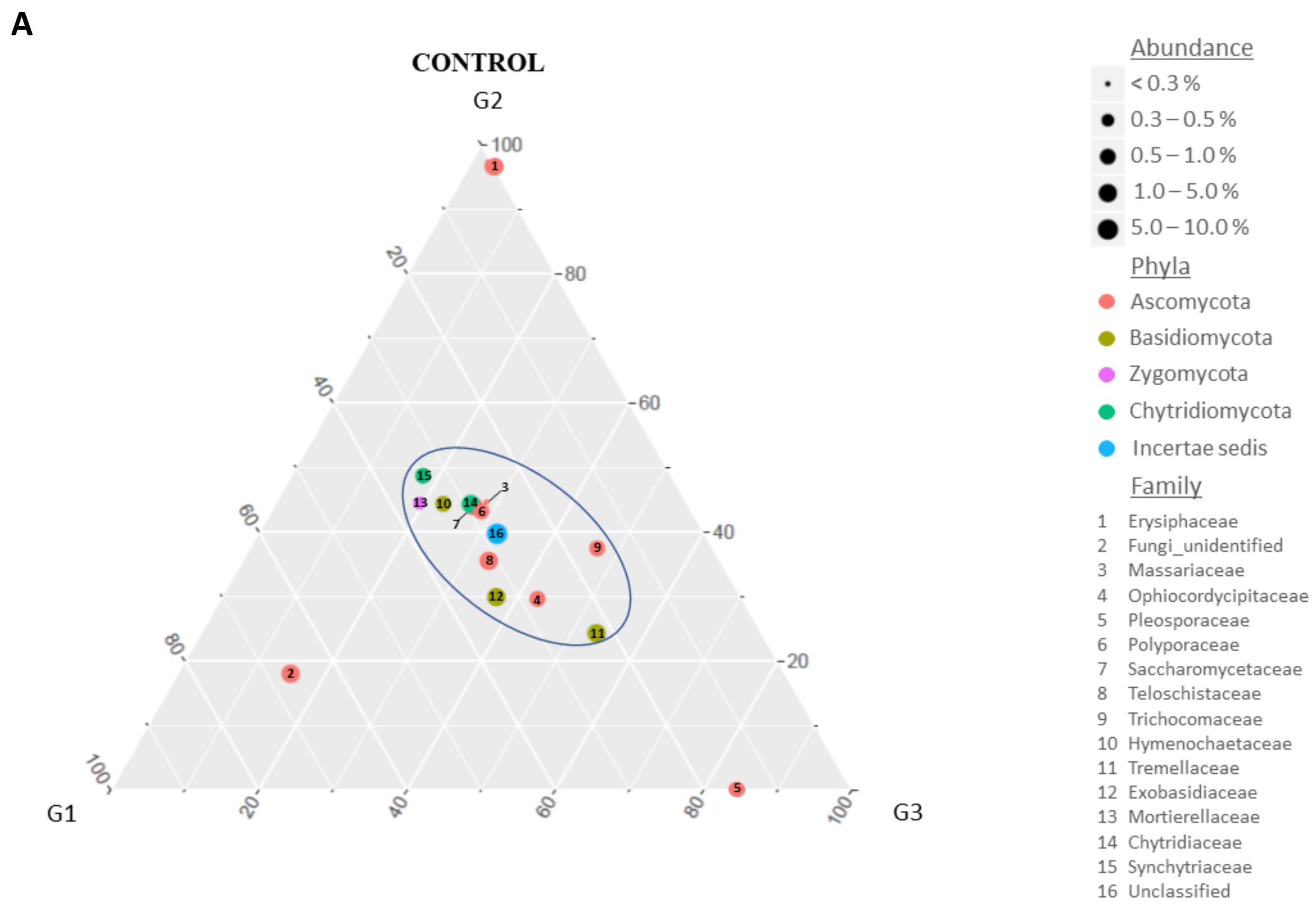
A
CONTROL
G2
G1
G3
100
80
60
40
20
Abundance
< 0.3 %
0.3 – 0.5 %
0.5 – 1.0 %
1.0 – 5.0 %
5.0 – 10.0 %
Phyla
Ascomycota
Basidiomycota
Zygomycota
Chytridiomycota
Incertae sedis
Family
1 Erysiphaceae
2 Fungi_unidentified
3 Massariaceae
4 Ophiocordycipitaceae
5 Pleosporaceae
6 Polyporaceae
7 Saccharomycetaceae
8 Teloschistaceae
9 Trichocomaceae
10 Hymenochaetaceae
11 Tremellaceae
12 Exobasidiaceae
13 Mortierellaceae
14 Chytridiaceae
15 Synchytriaceae
16 Unclassified

B

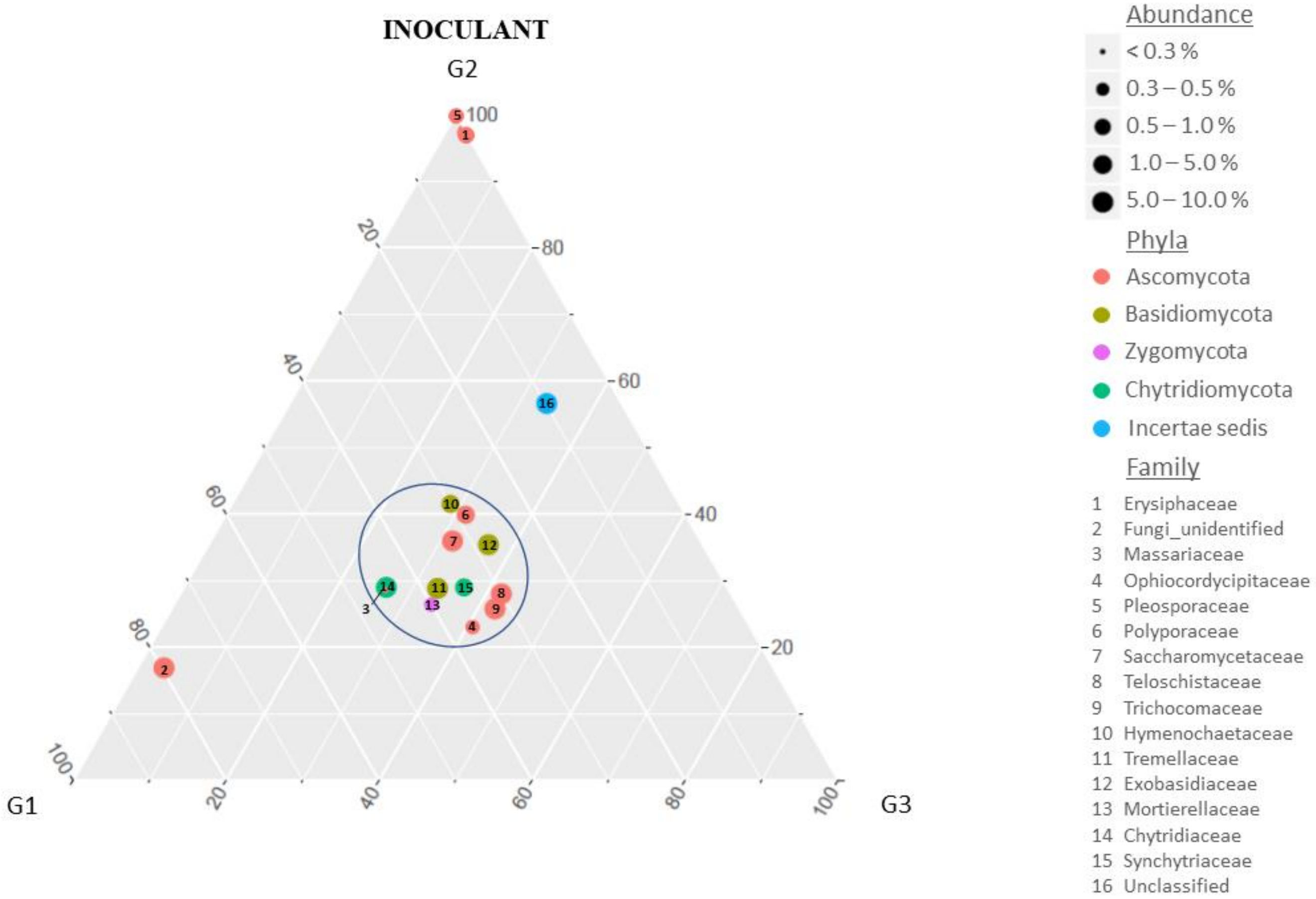

INOCULANT
G2
G1
G3
100
80
60
40
20
Abundance
< 0.3 %
0.3 – 0.5 %
0.5 – 1.0 %
1.0 – 5.0 %
5.0 – 10.0 %
Phyla
Ascomycota
Basidiomycota
Zygomycota
Chytridiomycota
Incertae sedis
Family
1 Erysiphaceae
2 Fungi_unidentified
3 Massariaceae
4 Ophiocordycipitaceae
5 Pleosporaceae
6 Polyporaceae
7 Saccharomycetaceae
8 Teloschistaceae
9 Trichocomaceae
10 Hymenochaetaceae
11 Tremellaceae
12 Exobasidiaceae
13 Mortierellaceae
14 Chytridiaceae
15 Synchytriaceae
16 Unclassified

C

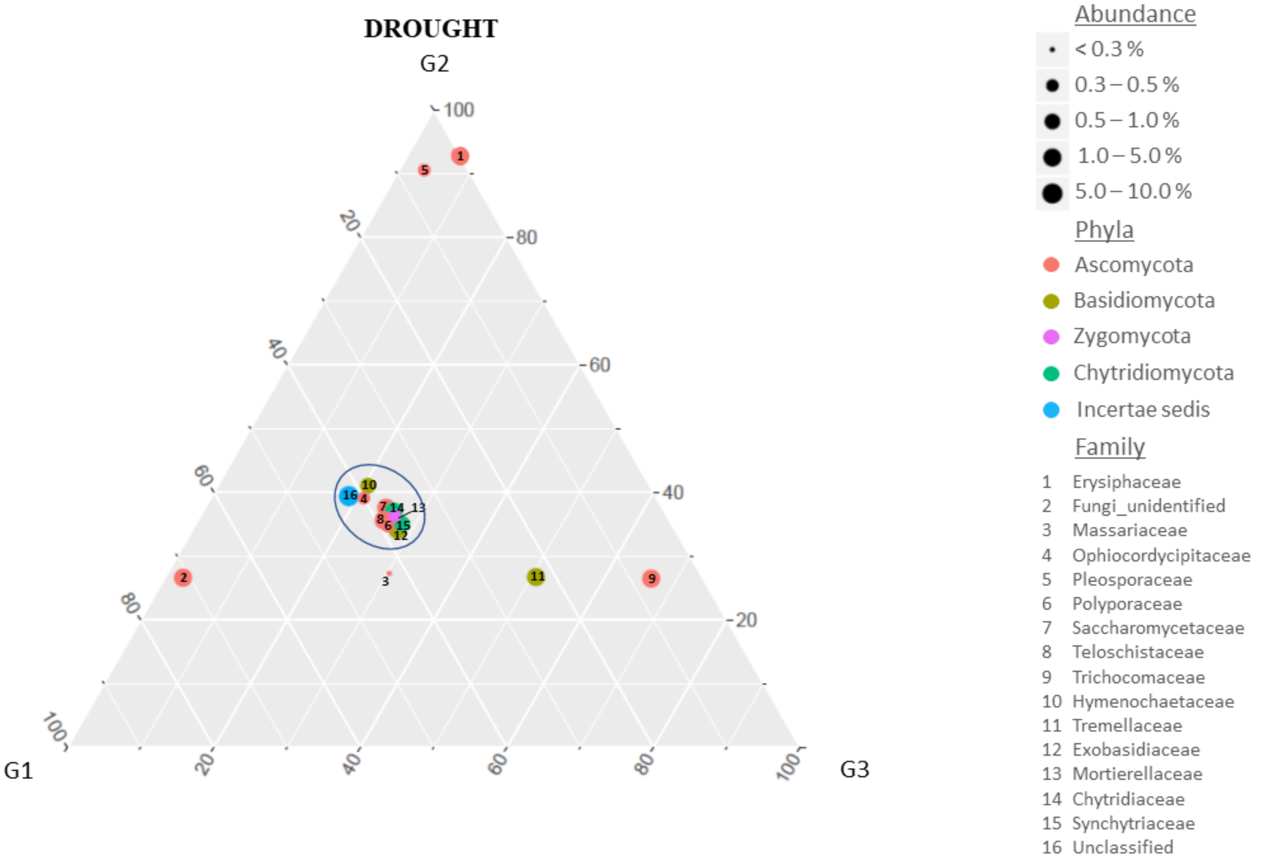
DROUGHT
G2
G1
G3
100
80
60
40
20
Abundance
< 0.3 %
0.3 – 0.5 %
0.5 – 1.0 %
1.0 – 5.0 %
5.0 – 10.0 %
Phyla
Ascomycota
Basidiomycota
Zygomycota
Chytridiomycota
Incertae sedis
Family
1 Erysiphaceae
2 Fungi_unidentified
3 Massariaceae
4 Ophiocordycipitaceae
5 Pleosporaceae
6 Polyporaceae
7 Saccharomycetaceae
8 Teloschistaceae
9 Trichocomaceae
10 Hymenochaetaceae
11 Tremellaceae
12 Exobasidiaceae
13 Mortierellaceae
14 Chytridiaceae
15 Synchytriaceae
16 Unclassified

D

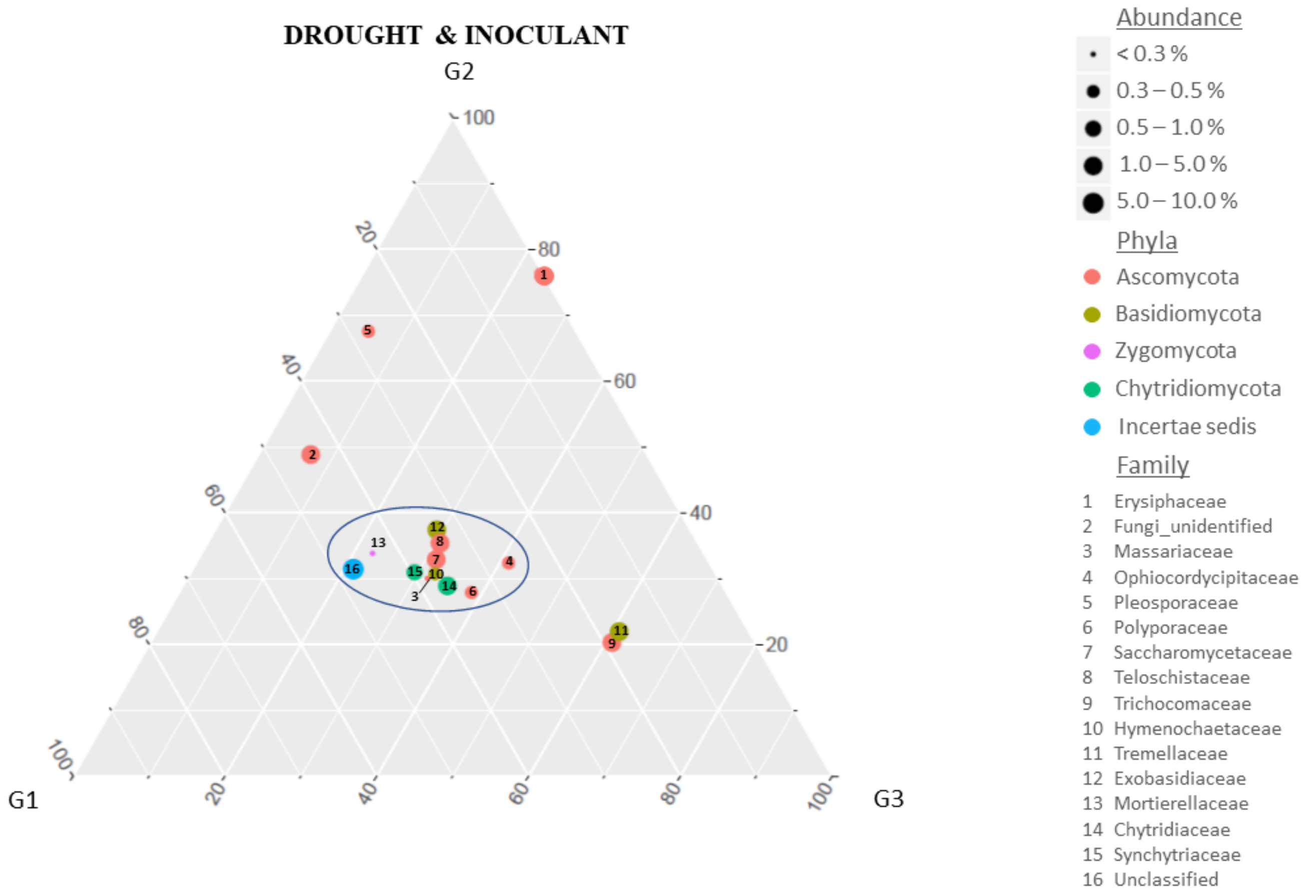


**Fig. 3S (A-D).** Ternary diagram showing the dynamic of sixteenth predominant endophytic fungal families in seed ecological niche-based on ITS reads across three (G1, G2 and G3) seed generations associated with **A.** standard-control, **B**. inoculant, **C**. drought, and **D**. inoculant plus drought treatments.

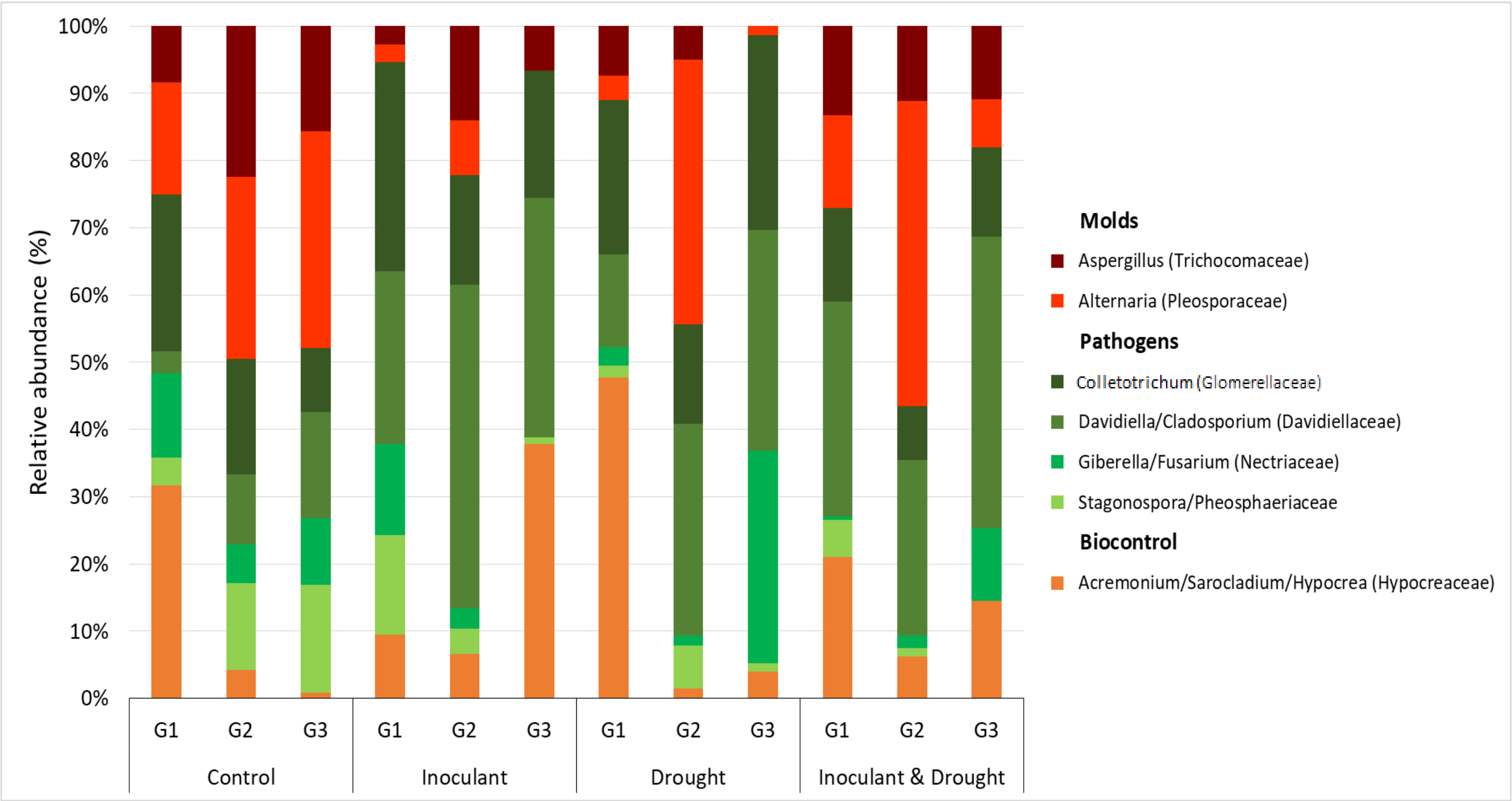


**Fig. 4S.** The relative abundance of the fungal OTUs classified as molds, pathogens and biocontrol in each G1, G2, and G3 generation of wheat seeds under standard-control, drought, inoculant, and inoculant plus drought treatment conditions.
